# Therapeutic relationships in aphasia rehabilitation: Using sociological theories to promote critical reflexivity

**DOI:** 10.1111/1460-6984.12590

**Published:** 2020-12-28

**Authors:** Felicity Bright, Stacie Attrill, Deborah Hersh

**Affiliations:** ^1^ Auckland University of Technology Auckland New Zealand; ^2^ Flinders University Adelaide SA Australia; ^3^ University of Adelaide Adelaide SA Australia; ^4^ Edith Cowan University Perth WA Australia

**Keywords:** therapeutic relationships, aphasia, rehabilitation, sociology, theory, critical reflexivity

## Abstract

**Background:**

Therapeutic relationships are fundamental in aphasia rehabilitation, influencing patient experience and outcomes. While we have good understandings of the components of therapeutic relationships, there has been little exploration of how and why therapists construct and enact relationships as they do. Sociological theories may help develop nuanced understanding of the values, assumptions and structures that influence practice, and may facilitate critical reflexivity on practice.

**Aims:**

To explore the potential for theoretical approaches from outside speech–language therapy to enable a deeper understanding of the nature and enactment of therapeutic relationships in aphasia rehabilitation.

**Methods & Procedures:**

An explanatory single case study of one speech–language therapist–patient dyad in an in‐patient stroke rehabilitation setting. Data included observations of five interactions, two interviews with the client and three interviews with the speech–language therapist. Analysis was guided by analytical pluralism that applied aspects of three sociological theories to guide data analysis and make visible the contextual factors that surround, shape and permeate the enactment of therapeutic relationships.

**Outcomes & Results:**

The analysis of this dyad made visible individual, interactional and broader structural features that illustrate the dynamic processes that practitioners and patients undertake to enact therapeutic relationships. Clinical practice could be viewed as a performance with each person continually negotiating how they convey different impressions to others, which shapes what work is valued and foregrounded. The patient and therapist took up or were placed in different positions within the interactions, each with associated expectations and rights, which influenced what types of relationships could, or were likely to, develop. Organizational, rehabilitation and individual practitioner structures assigned rules and boundaries that shaped how the therapist developed and enacted the therapeutic relationship. Whilst the therapist had some agency in her work and could resist the different influencing factors, such resistance was constrained because these structures had become highly internalized and routinized and was not always visible to the therapist.

**Conclusions & Implications:**

While therapists commonly value therapeutic relationships, social and structural factors consciously and unconsciously influence their ability to prioritize relational work. Sociological theories can provide new lenses on our practice that can assist therapists to be critically reflexive about practice, and to enact changes to how they work to enhance therapeutic relationships with clients.

**What this paper adds**

**What is already known on the subject**

- Therapeutic relationships are critical in aphasia rehabilitation. We have a good understanding of the different components of therapeutic relationships and how relationships are perceived by patients and practitioners.

**What this paper adds to existing knowledge**

- This study is novel in its use of sociological lenses to explore contexts and complexities inherent in building and maintaining therapeutic relationships. These are often invisible to the practitioner but can have a significant impact on how relational work is enacted and what forms of relationship are possible.

**What are the potential or actual clinical implications of this work?**

- This study will support clinicians to critically reflect on how they enact therapeutic relationships and may enhance awareness of the often‐hidden factors which influence the ways in which they work.

## Introduction

Therapeutic relationships are critical for patient outcomes and satisfaction in rehabilitation (Terry and Kayes 2019, Kayes *et al*. 2014). People with aphasia describe the importance of therapeutic relationships in rehabilitation, and there is growing evidence that these relationships influence patient experience, engagement and outcomes in aphasia rehabilitation (Bright *et al*. 2017, 2018a, Lawton *et al*. 2018a, 2020). There is some suggestion these relationships may be particularly important for those with more severe aphasia and/or those with greater rehabilitation needs (Lawton *et al*. 2019). Therapeutic relationships are valued and deeply embedded within the professional identity of speech–language therapists (SLTs) and within the discourses and socio‐cultural practices of the speech–language therapy community (Byng *et al*. 2002, Hersh 2010).

Kayes *et al*. (2014) argue that in rehabilitation, a therapeutic relationship involves activating the patient's own resources, moving the relationship from being an interpersonal connection to one that has a therapeutic effect. Therapeutic relationships do not happen by chance but require intentional action from the practitioner (Kayes *et al*. 2014, Bright *et al*. 2018a, Lawton *et al*. 2018b). In aphasia rehabilitation, strong therapeutic relationships require a holistic authentic relationship that recognizes and responds to the personhood of the client with aphasia (Bright *et al*. 2018a, Worrall *et al*. 2010, Lawton *et al*. 2018b, 2020). Relationships develop over time and require the therapist to be closely attuned and responsive to the patient (Lawton *et al*. 2018b, 2020, Bright *et al*. 2017). They are enhanced through emotional proximity and connectedness (Worrall *et al*. 2010, Lawton *et al*. 2018b, Hersh 2010). As such, therapeutic relationships are more than rapport between the practitioner and patient (Walsh and Duchan 2011), despite therapists often prioritizing rapport over relationships (Worrall *et al*. 2010).

The focus of empirical research on therapeutic relationships in speech–language therapy has examined what is done (or reportedly done) and how relationships are perceived, exploring the perspectives of people with aphasia (Fourie 2009, Lawton *et al*. 2018a, 2020, Hersh 2015, Bright *et al*. 2018a) and/or the perspectives and actions of practitioners (Bright *et al*. 2018a, Lawton *et al*. 2018b). However, how a therapist thinks and how they work are shaped by factors that people are not always consciously aware of. For instance, research on client‐centred practice in rehabilitation, of which therapeutic relationships are an important component (Terry and Kayes 2019), highlighted that it is affected by structural factors including time constraints and the clinical environment (Durocher *et al*. 2015). Similarly, in speech–language therapy, Foster *et al*. (2014) demonstrated how contextual factors influenced the prioritization of dysphagia over aphasia in stroke care. While Lawton *et al*. (2018b) identified that sociocultural factors (e.g., time pressures, length of stay and an emphasis on discharge planning) could latently influence the nature of therapeutic relationships, there has been little direct study of these factors. Such factors are commonly well entrenched and taken for granted (Paradis *et al*. 2019), making them challenging to ‘see’ in research that principally focuses on people's perspectives and experience of therapy.

Whilst research on therapeutic relationships commonly prompts participants to be reflective, it does not necessarily foster critical reflexivity, a process which prompts people to move beyond ‘simply’ reflecting on practice. Reflexivity requires people to monitor and manage practice as it is happening whilst reflection commonly occurs after the fact (Iedema 2011). Critical reflexivity also requires that they recognize assumptions, values and structures that underpin their practice and examine: (1) how these are enacted through daily practice; and (2) the effects these have, in particular the unintended consequences (Kinsella *et al*. 2012, Setchell and Dalziel 2019). This is challenging as discourses that surround and shape practice may be highly internalized, an ‘invisible cloud that pervades everyday life and everyday practice’ (Kinsella 2012: 46). It is important to understand the contextual influences on practice as there is a real risk that difficulties in building therapeutic relationships, or relationship breakdowns could be attributed to individual patients or therapists when, in fact, breakdowns could occur because factors surrounding the dyad influence what happens and what is possible. Using theoretically informed research approaches can help elucidate new perspectives that serve to unpack these influences on practice, and support practitioners to apply these new insights in reflection and practice (Thille *et al*. 2018, Paradis *et al*. 2019).

In this paper we use three theoretical perspectives to develop a multidimensional understanding of therapeutic relationships in aphasia rehabilitation, a form of analytical pluralism (Clarke *et al*. 2015). Analytical pluralism involves drawing on multiple theories or analytical approaches to develop rich, comprehensive understandings of a phenomenon that can enhance analysis derived from more traditional mono‐analysis (Clarke *et al*. 2015) (see the Methodology and Methods for more details). We examine an exemplar case of an SLT and a woman with aphasia using data gathered through a longitudinal study in stroke rehabilitation in New Zealand. This was previously analysed with a mono‐methodological framework (Bright *et al*. 2017, 2018a). By drawing on multiple theories that allow for new interpretations of the data, we seek to make visible the contextual factors that surround, shape and permeate the enactment of therapeutic relationships between people with aphasia and SLTs, providing richer understandings of therapeutic relationships. This approach has been successfully applied in other research in rehabilitation, for example, examining motivation in rehabilitation (Papadimitriou *et al*. 2018). Using multiple theories in analysis helped to identify how motivation is constructed through interactions with people and the rehabilitation environment. This work opened up different ways of thinking about motivation beyond the dominant understanding of it as an individual trait. Such an approach can highlight the complex and multidimensional nature of practice, reveal different possibilities for action and facilitate person‐centred care (Papadimitriou *et al*. 2018).

The specific aims of this study are twofold:

- To understand how the enactment of therapeutic relationships is influenced by factors that surround practice.
- To demonstrate how ‘thinking with theory’ (Jackson and Mazzei 2012) might enhance critical reflexivity.

## Methodology and methods

This study used an explanatory, single‐case‐study methodological approach (Baxter and Jack 2008) to enable a deep exploration of the interactions captured between Anna (an SLT) and Olive (a 70‐year‐old woman who had recently had a stroke). It sits within a constructionist paradigm (Crotty, 1998) which holds that knowledge is socially constructed and can be understood from multiple perspectives, influenced by the positions that the participants and researchers hold and are ascribed. We acknowledge our own positionality as three white middle‐class female, doctoral‐qualified speech–language therapy practitioner–researchers from Australasia. We are experienced in examining professional practices in speech–language therapy (Bright *et al*. 2018a, Hersh 2010, Attrill *et al*. 2020) and our work has often focused on what happens within interactions in dyads and how the parties experience these interactions. However, over time, we have become increasingly interested in how interactions, and experiences of interactions are shaped by factors beyond the therapeutic dyad. This led to this research, and to the selection of three specific theories that derive from different schools of thought that each provide a unique interpretative lens to the data. Our own positions, interests, experiences and privileges have shaped our interest in examining how relationships are constructed; they have also influenced the choice of theories used in analysis and how we interpreted the data.

We used three theories to guide our analysis, in which we examined and made sense of the contextual factors that influence the enactment of therapeutic relationships: Goffman's Dramaturgical approach (Goffman 1959), Positioning Theory (Davies and Harré 1990) and Structuration Theory (Giddens 1984). These theoretical perspectives are discussed in detail below (see the Results section). This form of analytical pluralism, using multiple theoretical perspectives, is rare in speech–language therapy but is increasingly used in qualitative research (Clarke *et al*. 2015) and in rehabilitation research (e.g., Papadimitriou *et al*. 2018) because it helps provide different and often more nuanced understandings of a phenomenon, and assists researchers to explore practice at multiple levels (Clarke *et al*. 2015, O'Leary *et al*. 2020). Using multiple theoretical perspectives enabled us to reveal the ‘pluralism of complexities that influence a phenomenon’ (Rogers 2012: 6). This form of analytical pluralism therefore helped us to add depth to our knowledge of therapeutic relationships (Rogers 2012). The three theories we have used were selected for several reasons. They allowed us to better examine how contexts might influence practice, responding to an identified gap in the knowledge (Bright *et al*. 2018a). They facilitated attention to the interactions between the different facets and people within therapeutic relationships: the client, the practitioner, the interaction between them and the broader context surrounding practice. While the three theories demonstrated commensurability with their consistent focus on communicative acts and meaning‐making (Clarke *et al*. 2015), the differences between them allowed us to highlight the complexities of practice, honouring and making explicit the complexities of everyday therapy (Clarke *et al*. 2015). They addressed relationships at different levels: the micro‐level of Goffman's work; Positioning Theory that connects communicative acts, individual's positions in interactions, and the narratives that surround interaction; and the broader perspective of Structuration Theory that explores how societal, institutional and individual structures guide actions and decision‐making. We acknowledge that we have used specific features of these theories in our approach to analytical pluralism; each theory in its entirety is complex and beyond the scope of this paper.

The data were gathered during an observational study of engagement in stroke rehabilitation. This examined how multidisciplinary practitioners worked with people experiencing communication disability (Bright *et al*. 2017, 2018a). The original study was underpinned by the Voice Centred Relational Methodology (Bright *et al*. 2018b). While findings suggested that practitioners were strongly influenced by the professional, disciplinary and structural contexts that they worked in, this methodological approach did not allow for in‐depth examination of these influences (Bright 2016). This current paper examines data from one SLT–client dyad. This dyad was selected from the 32 practitioner–client dyads in the original study as it demonstrated relational patterns common across many dyads in the research and the nature of the observational and interview data allowed for detailed examination of therapeutic relationships in rehabilitation.

### The case: Olive and Anna

#### Participants

Olive (a pseudonym) was a 70‐year‐old woman with aphasia following a left hemisphere stroke. The stroke primarily affected Olive's language. Her aphasia severity on the OWH Scales (O'Halloran *et al*. 2009), a series of rating scales for the severity of speech, language and cognitive communication impairments, was moderate on admission to rehabilitation, and mild on discharge. Clinically, Olive presented with mild receptive deficits and a moderate non‐fluent expressive aphasia, characterized by word‐finding difficulties. She had mild right‐sided weakness and reduced balance. She transferred to the rehabilitation ward 4 days after her stroke. Data collection started on this day. She had sessions with physiotherapy, occupational therapy and speech–language therapy during rehabilitation. Her physical issues resolved quickly during her admission. Olive was married with adult children and lived with her husband. Before retirement, she worked in social services. She was active in her community.

Anna (a pseudonym) was an SLT in her 20s who worked full time in inpatient stroke rehabilitation. She had approximately 5 years’ post‐qualification experience. She valued relationships with her patients, saying ‘building rapport and the therapeutic relationship is what I love the most, it's so important in therapy’. At the time of data collection Anna was supervising a student SLT, a new role for her. The student, who was not a participant in the research, was present in the observed sessions. The first session reported in this study occurred 5 days after Olive's stroke.

#### Context of the research

Olive was an inpatient in a 24‐bed neurological rehabilitation ward, offering rehabilitation for those over 16 years of age. The care of people over 65 years was overseen by a geriatrician. The first week of rehabilitation focused on assessment and goal‐setting as the service required goals and a preliminary discharge date to be set within this time. Each patient had an allocated keyworker, a nurse or allied health therapist whose role was to facilitate goal‐setting, communication between the team, patient and family, and discharge planning. As Anna was Olive's keyworker, some of the data captured Anna in her keyworker role. The service had an espoused rehabilitation philosophy, although the medical model of practice was often evident. Key decisions were made and communicated by the medical team. There were two consultant‐led ward rounds each week attended by the medical team and a nurse; on the other days, a junior doctor reviewed each patient. While discharge dates were often agreed in a weekly multidisciplinary team meeting, Olive's discharge date was set during a ward round before a team meeting was held, and was made with no input from the multidisciplinary team, or from Anna, whose role was to coordinate rehabilitation and discharge planning. The length of stay on the ward varied from 1 to 13 weeks. Olive's length of stay was 12 days.

#### Data collection

The data were transcripts and field notes of observations of interactions between Anna and Olive, and five transcribed interviews. Observations captured two informal interactions such as conversations in the dining room of the ward, two structured speech–language therapy sessions, and a goal‐setting/discharge planning meeting, attended by staff. The original observations were completed by the primary author. All were audiotaped and the two speech–language therapy sessions were video‐taped. Olive was interviewed after one therapy session and after discharge; Anna was interviewed after two therapy sessions and after Olive's discharge.

#### Data analysis

The transcripts were collated into one document by the primary author. Analysis involved all three authors in an iterative process of reading the transcripts, considering the case through each theoretical perspective and discussing the analysis in six group videoconferences. Each author focused on one particular theory and, indeed, on a specific component of each theory as detailed in the Results section. These functioned as ‘sensitizing concepts’ that informed what the author attended to within their analysis (Papadimitriou *et al*. 2018).

Each person reviewed the collated transcript asking theoretically informed questions of the data such as ‘what impressions is Anna working to give to others, and how does she do this?’ (Goffman's Dramaturgy), ‘what positions do Olive and Anna take up and how is this evident?’ (Positioning Theory), and ‘what structures are evident through Anna's practices and language?’ (Structuration Theory). Each person presented an initial analysis and the team discussed how the analysis differed when examining it from different perspectives. After each discussion, we individually returned to the transcripts and to the theoretical literature to each further develop the theoretically informed analysis. As analysis progressed, our meetings focused on specific incidents which provided rich insight into the factors influencing therapeutic relationships when viewed from the three different perspectives, such as those where the three approaches highlighted subtle but significant factors influencing how relationships were enacted. We focused on these in detail, each author examining the incidents from their chosen theoretical perspective before then coming together to discuss similarities and differences across the theoretical ‘findings’. Consistent with other theoretically pluralistic research (Papadimitriou *et al*. 2018), we did not seek consensus across the theoretical perspectives. Indeed, the very purpose of using different perspectives was to highlight the different ways of knowing and understanding practice and to illuminate the often‐hidden complexities inherent in practice (Clarke *et al*. 2015, Papadimitriou *et al*. 2018). These differences are apparent in how each theoretical analysis is presented in the Results section. We purposefully selected analytical findings that attended to the research aims, selecting specific components such as those that revealed the complexities of everyday, taken‐for‐granted clinical practice which impact on the enactment of therapeutic relationships.

#### Rigour

The data for the original case were collected as part of a PhD (Bright 2016) with interviews and field notes for methodological triangulation. Tracy's (2010) quality criteria guided rigour for both the primary study and this secondary analysis. These include worthy topic, rich rigour, sincerity, credibility and significant contribution. Such an approach is recommended for studies taking a pluralist approach to analysis (Clarke *et al*. 2015). In the Introduction we argued that this research addresses a *worthy topic*, that a focus on the factors influencing therapeutic relationships is timely and of value in advancing clinical practice. *Rigour* is demonstrated through engagement with the underlying theories, the close attention to source data, and a prolonged analysis approach which included peer checking with academics with experience in sociological analysis and presentations to SLTs at two international conferences. People's questions and feedback functioned as a form of member reflection (Tracy 2010) and helped identify areas where further clarity was required. *Sincerity* pertains to critical reflexivity and transparency. We have made our own positioning explicit and discussed this throughout the analysis process. Throughout our analytical discussions, which occurred over 3 years, we have discussed, debated and questioned our analyses. We have provided transparency about the research process through this paper. This also pertains to *credibility* that the results are trustworthy and plausible, shown through thick description, crystallization and transferability. Through the analysis we have provided detailed thick descriptions, interweaving both the data from interviews and observations along with the theories to ensure that readers could both understand the scenario and understand our theoretical interpretations. We did not seek to provide one answer for how therapists enact relationships; indeed, that would be contrary to our aim of showing how thinking with different theories could open up different understandings about practice. In presenting three theoretically informed analyses, we demonstrate crystallization, in which an analysis sheds a light on different aspects of a phenomenon (in this case, the enactment of therapeutic relationships). We intentionally chose an everyday case and supported this with details of the interaction to help ensure the case resonates with readers and allows them to transfer ideas from the analysis to their own practice. Finally, this work makes a *significant contribution* to speech–language therapy by extending our thinking about therapeutic relationships. It builds on existing research and responds to identified gaps in research (Bright *et al*. 2018a) by examining the broader external and structural factors that impact on practice. Integrating sociological theory also demonstrates methodological significance, extending dominant approaches to research within the field. Together, these steps demonstrate rigour (Clarke *et al*. 2015, Tracy 2010).

## Results

Each theoretically informed analysis is presented separately. We first present a micro‐sociological analysis focusing on the performances within the face‐to‐face interaction using Goffman's Dramaturgical approach, then examine the positions taken up, ascribed to, and resisted by the patient and practitioner within interactions, and finally present a broader structural analysis of the context and structures surrounding practice using Structuration Theory. In each analysis, we first describe the theory and then present the resulting analysis. The analysis contains descriptive summaries of care generated from the researcher's fieldnotes, as well as quotations from interviews and observations. Where direct quotations are integrated into the analysis, we use the following notations: IntAnna = interview with Anna; IntOlive = interview with Olive; Obs = observation of both Olive and Anna; and ObsAnna = observations of Anna in a team meeting.

### Exploring the performances of relationships using Goffman's Dramaturgical approach

#### Goffman's Dramaturgy

Erving Goffman looked at the subtle details of how people behave in the presence of others, that is, of face‐to‐face interaction in everyday life (Goffman 1959). His ideas were influenced by symbolic interactionism, a notion of dynamic interplay where people negotiate their sense of self through an iterative process of presenting themselves to an audience who receive and react to that presentation, thereby shaping further reactions in turn. In this paper we use concepts from Goffman's *dramaturgical approach* that claims that face‐to‐face interactions are like a performance. Goffman suggested everyday interactions involve a *front stage* where a person is on view and acutely aware of the need to manage their performance in front of another; and a *backstage*, a private place to practice or prepare for the front stage. This idea of the front stage with its ‘tone of formality’ (Goffman 1959: 78) being quite separate to the backstage characterized by informality found in many contexts but is not always clear cut:

team‐mates with respect to one show will be to some degree performers and audience for another show … activity in a *concrete* situation is always a compromise between the formal and informal styles by reference to backstage and backstage activity. (79)

Goffman suggested that we actively work to portray ourselves in the best light, and that we have different *masks* for different audiences. This contributes to *impression management*, a notion that captures the work we do to portray ourselves carefully to others on the front stage. Important elements for impression management include setting, appearance and manner (behaviours). Goffman distinguished between information that actors *give* to their audience (verbal and non‐verbal elements that are consciously used) and information that they *give off* (elements that are unconscious and unintended). These ideas have been applied to explain how health professionals ‘perform’ in their work environments including in acute hospital settings (Lewin and Reeves 2011) where the actual space used by different professions and audiences can shift between front and backstage, and where there are both planned and ad hoc interprofessional interactions in both front and back regions.

Our analysis examines two front‐stage performances evident in the interactions between Anna and Olive to illuminate and explain the nature of their relationship. We consider Anna's presentation as a relational therapist, and Anna's performance as a member and representative of the wider team and the service.

#### Anna's presentation of self as the relational SLT

When Anna and Olive met for the first time, they did so in a shared lounge and dining area. Anna had her student with her. Anna dressed professionally, but not overly formally. The *look* of her clothing contributed to a particular impression of her as a health professional, but also as an approachable, authentic person—her *front‐stage* presentation. Other than another sleeping patient and the researcher, the three were alone in this room. They sat together on a lounge suite: Anna on a chair and Olive and the student on the sofa. There was no table or desk between them. Considering this setting through Goffman's lens of the front stage, there appears to be a controlled informality about this encounter, and a portrayal of Anna as deeply engaged in the encounter. Anna focused her attention on her interaction with Olive and when the student worked with Olive, Anna maintained her attention towards what was going on, taking notes unobtrusively, making occasional comments, nodding and smiling throughout the session.

Despite this performance, the researcher picked up on the subtle messages that Anna *gave off* rather than *gave* around the presence of the student, and raised these with Anna later. Anna described a tension in being a supervisor that it was ‘bloody hard’ (IntAnna) to sit on her hands while the student was working with Olive. She said that her style was more chatty and responsive than the student's style, and that making small talk and some self‐disclosure helped develop a connection with patients. Anna did not consider rapport‐building to be a strength of her student and later commented that her lack of opportunity to set the stage of the relationship affected her ongoing ability to work productively with Olive.

I think a lot of the information I tried to dig in the initial sessions was more difficult to kind of establish because I had more of a hands off role and I didn't want to prompt the student too much to do what I would do. (IntAnna)

Nevertheless, Anna made a conscious effort in her self‐presentation *front stage* during sessions, and actively considered how Olive might perceive aspects of her manner in a way which would be conducive to the therapeutic relationship:

I try to chuck in little remarks that release the pressure of trying to communicate, trying to break the awkwardness by letting her participate in something she can, something we can both do—telling a funny or acknowledging that it's me that isn't getting it. … As much as I want to get the (assessment) info, I think it's more that I think the patient feels I'm putting an effort into getting to know them, to connect with them at a *level* where they can participate and get the message across. (IntAnna)

This quotation captures Goffman's point that much of what people do in their performance is not simply to achieve a goal (in this case, acquiring information about the patient or completing assessments) but to manage how they are perceived by others, and how what they do can affect what the other person then does in return. Anna was working hard on impression management in this session. Despite feeling dissatisfied with her student's ability to run the session as she would have, Anna did not show this overtly in the session. Moreover, what she chose to reveal to the researcher afterwards would also have been carefully managed (whether or not consciously).

#### Anna's role as representative of the wider team and service

Anna talked about the importance of relationship building in her interview. However, she appeared to be balancing this with her professional need to move through her ‘checklist’ (IntAnna), to do the assessment and goal‐setting expected of an SLT with a new client with aphasia. Anna's audience was not only Olive but also her student for whom she needed to appear efficient and knowledgeable, and her colleagues who expected her to complete particular actions. Additionally, as Olive's keyworker, she had a more complex role in coordinating Olive's care. Goffman (1959) recognized that people working within a team, or for an agency or employer, project beyond themselves as individuals to themselves as representative: ‘One finds that service personnel … enliven their manner with movements which express proficiency and integrity, but whatever this manner conveys about them, often its major purpose is to establish a favourable definition of their service or product’ (47). In this context, Anna needed to retain a positive performance for Olive. However, her interview suggested that the decision about the discharge date, made without her input by the medical team, undermined her role. This suggests that she may have adopted a front stage performance with her team colleagues, maintaining composure despite feeling that she was not properly consulted. Equally, the discharge plan impacted on her continued efforts to engage with Olive as she would have liked:

With Olive, I do feel like she told *me* what she wanted to tell me and nothing more. Which is fine … but I felt like I only got a snapshot of what she was. And I think had she of been with us longer and had I been the lead clinician that I potentially would have been able to get a bit more information, maybe? She seemed like a relatively private person …. (IntAnna)

Anna's assessment of Olive as a ‘private person’ is part of her explanation to the researcher about why she was only able to achieve ‘a snapshot of what she was’ (IntAnna). This admission reflects Anna's dissatisfaction with the lack of depth in the relationship with Olive considering her desire to be viewed as working relationally. Anna had to adapt her performance on the front stage in response to several factors: the presence of her student and need to take a supervisory role; Olive's request to go home impacting on Anna's ideal therapy plan; and Anna's need, as key worker, to represent the team discharge decision positively even though she was not party to it. The overall effect of this was that Anna became more process oriented and more distanced from Olive. As she said, ‘had I been the lead clinician that I potentially would have been able to get a bit more information …’ (IntAnna). We interpret this to imply a closer relationship with more openness and trust, aspects which underpin an effective therapeutic relationship.

#### How a dramaturgical approach helps us understand therapeutic relationships

Goffman's theories about interactions as performances, as careful impression management, are helpful in understanding how Anna *played a part* within the *drama* of the rehabilitation context, carefully managing the impressions that she gave to her audiences at any particular time and adapting in response to their reactions. While an assumption could be made that Anna's performance was front stage for her patient and backstage with her colleagues, this case suggests a more fluid set of performances, varying with different team members. A dramaturgical perspective allows a micro‐level view on exchanges as well as a broader theoretical view about how people try to control how they are perceived by others. This micro‐level view highlights what we take for granted in the way we manage setting, appearance and manner.

### Explicating the positions ascribed and taken up by individuals within interactions

#### Positioning theory

Positioning theory examines how rights and duties to speak and behave in particular ways are distributed by participants within interactions (Davies and Harré 1990) ‘Positioning’ refers to the interactionally mediated process of assigning people to different positions; ‘positions’ refers to the metaphorical place(s) or space(s) one holds within an interaction. These places/spaces are accompanied by particular attributes, rights, duties and expectations, many of which are implicit (van Langenhove and Harré 1999, Harré 2015). Whilst positions might be associated with attributes or roles (e.g., viewing oneself as ‘person‐centred’, or being an SLT), they are never fixed. Instead, they are dynamic, constructed and enacted through interactions (Davies and Harré 1990). Positions are expressed in the storylines, the patterns of ‘being’ that people create and enact through verbal and non‐verbal communicative acts. Storylines, communicative acts and positions are at the centre of an analysis using Positioning Theory. People can intentionally or unintentionally position themselves (‘reflexive positioning’) and also position others (‘interactive positioning’). *Reflexive positioning* refers to the process through which people position themselves privately in their thoughts or through internal discourse (e.g., seeing themselves as an expert in a particular area), whilst *interactive positioning* where someone positions others through their speech or other communicative acts (e.g., where one is described as an expert by others, or where others defer to their opinion, waiting for them to speak and nodding in agreement) (Davies and Harré 1990). People can resist positions that are ascribed through interactive positioning. Issues of power and vulnerability are innately entwined in the process of positioning; it is through implicit or explicit reference to power and vulnerabilities that people are able to ascribe positions to others (Harré 2015).

This critical analysis examines how, through interactions, both Olive and Anna took up, were ascribed and resisted different positions. Each of these influenced how they were able to develop a therapeutic relationship, and indeed, what the nature of that relationship was. The positions presented here are not exhaustive; instead, they illustrate different positions that people may hold in rehabilitation which can impact on what relational possibilities may exist.

#### A compliant patient and rehabilitation facilitator and expert

Olive was admitted to rehabilitation 5 days after her stroke. Stroke was not something she had experienced before; this experience, and the specific challenges she was having such as aphasia and fatigue, were new to her. In this situation, it is not surprising that Olive, or any patient, would take up the position of a ‘compliant patient’, one who ‘goes along with’ the rehabilitation processes led by the rehabilitation practitioners. This was evident in her agreement to take part in assessments and therapy activities and her active participation in assessment sessions (Obs).

As a ‘compliant patient’, Olive's role was to do what she was asked to do. This is not to say she was coerced into this role or only took up this role because of Anna's actions. Olive described wanting to do therapy so she could improve; this suggested she may have taken up the role of ‘compliant patient’ through a process of *reflexive positioning*. However, this position was reinforced through Anna's actions, a form of *interactive positioning*. One such example was coming into Olive's bedspace and setting up the session before seeking her consent for this session. Anna came into the space followed by her student and a rehabilitation assistant. As she was asking Olive if it was convenient to have the session, Anna started moving furniture and Olive's belongings around to create space for the three people—herself, the student and the assistant (Obs). Olive's *duty* as patient was to agree to participate in the session. These actions conveyed a sense of Anna holding some control in this environment, consistent with a position of being a ‘rehabilitation facilitator and expert’, although this expertise was mediated through the small talk that followed, including asking how Olive was feeling and how she slept (Obs). This reflects the *storyline* (the internal narrative) that underpinned Anna's practice. This had two coexisting components: first, her valuing of relationships, enacted through actions such as small talk and empathetic attentiveness to Olive in comments such as ‘I don't know a lot about you … I would like to get to know you a little bit’ (Obs), and evident in her descriptions of her own practice such as ‘building rapport and the therapeutic relationship [is] what I love the most and see as so important in therapy’ (IntAnna), and second, her focus on completing rehabilitation tasks and processes such as completing assessments and goal‐setting:

I have a few other questions to ask you. I know this is hard work but you are doing really well and I am getting lots of information from this and it's excellent. … Just a few things we look at before we send people home … just making sure that the little things in life flow well. (Obs)

Olive's position of ‘compliant patient’ was reinforced through the rehabilitation practitioners’ expectations. Participating in ‘normal’ activities was expected, with Anna describing Olive's *duties*, telling her ‘They will be roping you in to something called breakfast group I would say. … They will be making you make your own breakfast’ (Obs). Later Olive described going to an ‘education session’ on the ward saying she didn't know it was happening, but she was told to go (IntOlive). Participation was expected as part of being a ‘compliant patient’. This was seen in Anna's description of the rehabilitation timetable, a therapy schedule that was placed on the patient's wall. Anna said:

So it will pop up … there is a fairy that comes around and writes it up on your timetables in the morning and you just sort of look at your timetable ‘oh I've got speech at this time’. … It will be written on your timetable so you will know when to expect us.(Obs)

In the position of ‘compliant patient’, she had a *right* to rehabilitation, but there was a coexisting *duty* to wait for when the therapist was available. She was left ‘not‐knowing’ when therapy would happen. This was normal practice on the ward; Olive was expected to accept this. She was expected to be compliant *and* patient. Olive could not appeal this without breaching the ‘norms’ expected of a patient. Likewise, when Anna asked if it were ‘okay to start [the session]’ (Obs) in the example above, this was a rhetorical question which Olive, as ‘compliant patient’, could not decline without breaching the *duties* expected of her. Olive's position as ‘compliant patient’ was intimately entwined with Anna's position as ‘rehabilitation facilitator and expert’, someone experienced in stroke rehabilitation who determined what was done, when and where, something that gave her power over Olive (Dagg and Haugaard 2016).

#### A resistant patient and compliant therapist

While Olive was positioned in, and initially took up the role of ‘compliant patient’, throughout the first week it became apparent that Olive was resisting this positioning. She took an active role in decisions about her care, telling doctors she wished to go home (IntOlive). Anna said the ‘medical team jumped on [Olive's] desire to go home’ (IntAnna). This may have been prompted by Olive's dissatisfaction with rehabilitation: ‘I don't want to be here so I think maybe (gestures leaving)’ (IntOlive). A factor in this appeared to be the lack of therapy input: ‘Well, I think I would be (indicating going from one thing to the other) but only little things, little bits’ (IntOlive). In telling the doctors that she wished to be discharged, she resisted the role of ‘compliant patient’ and instead started to position herself as a ‘resistant patient’, another example of *reflexive positioning*. While Anna had stated that Olive would have therapy ‘every day … and what will potentially also happen is you will end up having speech therapy twice a day’ (an espoused *storyline* of intensive interactions and therapy input), this did not happen. The lack of therapy (which served as a form of communicative act) challenged this storyline, and subsequently Olive's positioning. Through her communicative acts, she resisted the expectation that she would wait for therapy to be delivered according to the practitioners’ schedule and took a more assertive, self‐advocating role. Engaging in advocacy and resisting dominant positioning may be a familiar position, given her former professional role in social services.

The medical team accepted Olive's request and directed the team (especially Anna as Olive's keyworker) to organize discharge (IntAnna). As a result of Olive's resistance to her ascribed position as a ‘compliant patient’, Anna was ascribed the role of ‘compliant therapist’, compliant to the needs and directions of the patient and the medical team. This position was somewhat in tension with Anna's reflexive positioning as a relational therapist. In an interview after Olive was discharged, Anna described how she ‘changed the plan of attack’ to ‘become more task focused’ (IntAnna). This saw her ‘disengage from [the] role of being therapist … you become a bit more matter of fact … and say “ok this is what is happening and we need to really think about what is going to happen and what needs to be done”’, becoming ‘a lot more tick boxy’ (IntAnna). Of note, Anna was positioned not just through Olive's actions and the medical team's directions; she also needed to comply with the spoken and unspoken expectations of her colleagues in the community stroke team who would provide Olive's rehabilitation at home. She described previous experiences:

In the past I have had a lot *of* feedback from [the community stroke team] where patients have gone home, especially when I was a newer therapist and patients have gone home and there's been boxes that haven't been ticked … so I guess you become more task focused than patient focused. (IntAnna)

This demonstrates how someone's position can be influenced by contemporaneous and historical communicative acts, and that that storylines (such as ‘what must be done before discharge’) can hold over time. Her colleagues’ expectations of Anna, the duties they expected of her, positioned her as compliant to them.

As a result of the positioning shifts, the relationship between Anna and Olive moved from one where Anna foregrounded developing a personal–professional connection to a relationship centred on facilitating discharge. These changes in positions meant Olive, because of her positional shift, exerted some power over Anna (as did Anna's colleagues), and the purpose, nature and length of their relationship was changed. This demonstrates how positions are dynamic and continuously renegotiated through interactions (Dagg and Haugaard 2016, Davies and Harré 1990).

#### How Positioning Theory helps us understand therapeutic relationships

Looking at the positions that both Olive and Anna were ascribed, resisted and took up, we can see different ways of viewing the relationship between the dyad. Taking this perspective prompts the practitioner to consider what types of relationship are *actually* possible and enacted; these may not match the relationship that client or practitioner *aspire* to. It highlights how ‘usual practices’ may unwittingly emphasize patient compliance and practitioner expertise, which creates particular relational configurations. This provides some insight into why espoused practices such as person‐centred care can be challenging to truly embed. This analysis demonstrates how the positions one takes, or are ascribed, are not simply those of one's conscious choosing. Instead, they are created, shaped and renegotiated through interactions and social contexts.

### Examining how social structures influence relationships using Structuration Theory

#### Structuration Theory

In his Structuration Theory, Giddens (1984) suggests that human agency is interrelated with the social structures of society and institutions. The actions of humans reproduce, but can also change the rules, resources and systems that include our traditions, institutions and cultural norms. These social structures provide established, largely internalized ways to act that we reproduce by repeating the associated actions. However, we can also actively change structures if we modify, replace or omit actions that are usually informed by the structure. In an inpatient rehabilitation setting, there are many such structures that guide how practitioners and patients act, the rules they adhere to and the roles they perform.

We monitor our actions in response to structures by examining the outcomes, and how surrounding factors influence these. In response, we may reflexively act outside of the usual rules, and these new actions can modify the structures. These new actions may be reproduced by others, becoming routinized and recursive, thereby creating new social structures. Therefore, social structures guide our actions, but we can also reflexively modify and produce new structures. This ‘structuration’ provides an interface between human agency and the social structures that guide our actions, which enables social systems to both continue and to change.

#### Structures guide Anna's relationship and practice with Olive

Anna's relationship with Olive was enacted through, and guided by, typical social practices of her inpatient rehabilitation setting that are structures that predict the actions of practitioners and patients. Anna had ‘knowledgeability’ about these structures which were stored as memory traces to guide the language used, rules about who does what and procedures germane to the setting. These structures guided Anna's enactment of everyday work tasks and decisions, informed her about Olive's situation, and helped her predict the actions to take and possible outcomes from these actions. There are three memory trace modalities: signification, legitimation and domination, that each provide structure for Anna's actions, but are also less visible to Olive. Anna and Olive's relationship can be interpreted using these modalities.

The first modality, ‘signification’ refers to the meaning encoded in language. The language concepts and semantic codes used in rehabilitation provided a schema for how Anna enacted her relationship with Olive. This language, expressed in terms such as ‘assessment’, ‘discharge’ and ‘discharge planning’, ‘returning to what you used to do’ and ‘what goals do you want to achieve?’ (Obs), assisted Anna to interpret events, make sense of rehabilitation activities and predict the outcomes of her actions, those of Olive and of her team members. Anna's knowledgeability about the rules and actions that relate to each term also set cognitive boundaries that both informed and limited her practice. For example, in the following quotation, Anna demonstrates knowledgeability of what *‘assessment*’ is, and what it is not, that enabled her to understand how she would enact assessment with Olive, and how this related to later rehabilitation activities.

We assess all of those things *just to make sure that we are picking up on anything that might be a little bit tricky*. So some of these tasks might be a little bit easy, some might be a bit harder. *So we are just going to continue* with this assessment. (Obs)

This quotation also suggests that Anna used language structures to control her relationship with Olive through revealing only certain information about how the assessment would unfold. Olive, conversely, relied on Anna to use language to reveal her actions and the processes of assessment. These signification structures relate to mental schemas about rehabilitation and organizational processes and hierarchies that Anna used to manage her relationship with Olive and predict practice outcomes, but which Olive had less knowledge about.

The second modality is ‘legitimation’, where Anna's practices were encoded in the norms and values of the rehabilitation team and setting. Anna's relationship with Olive was informed by legitimation structures that directed the rules and routines she followed as a rehabilitation team member. For example, Anna's assessment practice with Olive was guided by her practised knowledge of assessment processes, learned and repeated over again with many patients in the rehabilitation setting.

It's really important information for us to know when you do go home that … we are prepared for if there is something difficult that what we need support with. *So this assessment process can be quite time consuming. But it's quite important*.(Obs)

Anna rarely challenged these normalized processes; they appeared deeply internalized as part of her rehabilitation schema. Any deviation in the routines of Anna's practice, for example, to respond to Olive's expressed need for more speech and language therapy, would challenge these deeply embedded legitimation structures. For example, in the following quotation Anna prioritized completing the *‘discharge*’‐related processes she perceived as critical for Olive to function at home. She acknowledged that this changed the nature of and available time to enact her relationship with Olive. However, for Anna, these ‘discharge’ practices were part of an institutional rehabilitation routine that was legitimized, reproduced and deeply embedded within her practice schema.

But you disengage from your role of being therapist I guess. Not therapist but doing therapy. And I think you *become* a bit more matter of fact with a patient and *say ‘ok this is what is happening and we need to really think about what is going to happen and what needs to be done.’*(IntAnna)

Whilst rehabilitation structures have a functional, person‐centred intent, Anna also used these to ‘signal rationality’ to meet the expectations of ‘important others’ in the rehabilitation setting, including the broader team, hospital administrators and community providers, as well as those of Olive and her family. This reflects that Anna and Olive's relationship was therefore also directed by structures that legitimized forward moving and goal‐driven language that projected ‘efficiency and rationality’ to the ‘important others’. This legitimation of rehabilitation practices to meet competing expectations of several stakeholders in turn, sanctioned the nature of, extent and limits of Anna and Olive's relationship, and what was visible to Olive. This challenges Anna's assertion that the therapeutic relationship and patient‐centredness principally guide her rehabilitation practice.

In the final memory trace modality, Anna's rehabilitation practice could be interpreted as part of a ‘domination’ structure that allowed her to use power to manage resources and her relationship with Olive. This was evidenced when Anna used her key‐worker role to direct the frequency and nature of the speech–language therapy service, Olive's encounters with other practitioners, and to prioritize the completion of discharge related work.

Her discharging as quickly as she did, the focus changed from therapy to what do we need to do right now to make her be able to go home and to be able to function *and a lot more background work was* done, but that was not directly done through her. (IntAnna)

Anna prioritized domination structures that guided how she allocated time and therapeutic resources to Olive, even when Olive identified that she wanted more speech–language therapy. This demonstrates how Anna's actions reproduced deeply embedded social practices of the rehabilitation setting that directed her role as the practitioner with power and resources, and Olive's role as the recipient patient.

Giddens’ (1984) ideas about the three memory trace modalities illustrate how Anna managed her actions and interactions within the rehabilitation setting. Through practice and repetition, Anna had become knowledgeable about the rehabilitation rules and processes that guided her actions with Olive, and these were routinized. Whilst these structures predicted Anna's actions, Giddens also suggested that Anna could modify certain structures by taking alternative actions to those expected. For example, Anna demonstrated ‘reflexive awareness’ of the impact of her competing key worker and SLT roles on her relationship with Olive:

That's frustrating cos *there have been snapshots when I've seen the ideal but very rarely do I get to do that*. Not very rarely does it go in my thought process ‘cos I'm in my tick *tick* tick—I've done assessment, now I do therapy. (IntAnna)

To deviate from structures such as the rules that guide assessment, goal‐setting and discharge is effortful, as they are deeply routinized and rarely questioned. Implicitly for Anna, modifying these practice structures may also have had negative consequences for her position and power within her team. For example, if she had prioritized Olive's therapy instead of enacting discharge processes, these new structures may have disrupted the team's routines and her standing within the team.

#### How Structuration Theory helps us understand therapeutic relationships

Social structures provide us with the rules, routines and processes that help us to predict and feel secure about the actions that we take as practitioners enacting therapeutic relationships. Structuration Theory provides a broader view of the social practices of rehabilitation that are constructed and repeated by the rehabilitation team, individual practitioners and, to a lesser extent, the patients. Practitioners store these structures as deeply embedded memory traces that become the recursive language, resources and routines of rehabilitation and guide how therapeutic relationships are enacted and managed. Whilst these practice structures are rarely questioned and are not transparent to patients, opportunities exist for practitioners who reflect and act reflexively to change and produce new structures that direct different routines, practices and outcomes.

## Discussion

This analysis highlights some of the complexities inherent in building and maintaining therapeutic relationships and suggests that rather than simply ‘building rapport’, we and our patients are in a dynamic process of relationship building and breaking in a context of other relationships and structures. In many ways, clinical practice is a performance, with each person renegotiating how they convey different impressions to others. Anna's practice was strongly influenced by her relationships with her student and her team, as well as her patient. This was evident in her seemingly subconscious need to present herself carefully and deliberately depending on her role (therapist, educator, key worker, colleague) suggesting she spent a lot of her time in front stage performances, even for the interviewer in this study. Our analysis suggested a fragility to the relational work which she held to be a core component of rehabilitation because it was constrained by decisions beyond her control. Through interaction, Anna and Olive were both ascribed and took up particular positions that influenced what types of relationships could develop. This reflects how practice and relationships are co‐constructed, influenced through the communicative act of others. However, beyond these, the analysis also demonstrated how people's actions were influenced by internal and external structures, placing rules and boundaries around how the therapeutic relationship was enacted. Anna's way of working was not just influenced by what *she* valued, but by what was valued by colleagues, the rehabilitation model, and the organizational structure and expectations. Whilst the purpose of analytical pluralism is not to produce a single cohesive analysis, bringing together the key points from these three analytical approaches reveals differently nuanced understandings of relational practices as compared with the original interpretive analysis of these data which described the practices used to engage people in rehabilitation (Bright *et al*. 2018a), a form of crystallization (Tracy 2010).

Our multidimensional analysis represents a form of critical reflexivity, which helps us to understand the shaping of everyday practices (McCorquodale and Kinsella 2015, Kinsella *et al*. 2012). By unpacking the taken‐for‐granted rules, habits and traditions, we may be able to envisage new possibilities for action (McCorquodale and Kinsella 2015, Thille *et al*. 2018) and reduce the gap between work‐as‐desired, work‐as‐imagined and work‐as‐done. There is a growing move to promoting critical reflexivity in clinical practice, for instance, to interrogate therapeutic relationships in occupational therapy (McCorquodale and Kinsella 2015) and through the use of theory to examine care in paediatric neuromuscular clinics (Thille *et al*. 2018). We hope this paper might help SLTs not only to reflect on their ways of enacting relationships in practice, but also to start to ‘think with theory’ (Jackson and Mazzei 2012) and embed critical reflexivity along with the more common process of reflection‐on‐action (Kinsella *et al*. 2012). We suggest that these theories may provide useful frameworks to facilitate critical reflexivity (McCorquodale and Kinsella 2015), helping people look beyond what they do to why they work in this way, and what they achieve through their actions.

The three theoretical perspectives we used can prompt SLTs to ask different questions about their practice. Goffman's theories about interactions and performances might prompt practitioners to reflect on the parts they play and the audiences to whom they are performing. They prompt attention to potential tensions that arise when performing to different audiences, and challenge us to reflect on how our performances on the ‘front stage’, perhaps with a patient might differ from front or backstage performances with students or colleagues. These ideas can help us examine why we do this and how it reflects on us professionally and relationally. Positioning Theory might prompt practitioners to consider what positions they *expect* the patient to take, what rights and duties they associate with this, and how they interpret ‘breaches’ of these positions. They can ask what positions are *actually* ascribed and taken up by both the patient and themselves, and consider how each person's verbal and non‐verbal communication influences the positions people take up. Finally, Structuration Theory prompts practitioners to reflect on how therapeutic relationships, and indeed, our everyday practices are enacted through language, resources, routines and traditions. It challenges us to contextualize our understanding of practice, and to identify the recursive structures that underpin and guide what we do. Structuration Theory makes these visible, encouraging reflection about how structures direct and influence our therapeutic relationships and that we can be active agents who can modify the structures through positive, responsive actions. Through critical reflexivity, practitioners will be better equipped to actively question existing practice and help challenge and change the systems and structures that surround practice.

This study intended to demonstrate how different theoretical lenses might provide new insights into how therapeutic relationships are enacted in aphasia therapy, and how these are influenced by implicit and explicit internal and external factors. We used real, quite ‘run of the mill’ data from interactions between one practitioner and a person with aphasia in an early rehabilitation context. It is likely that the interpretation would vary for interactions derived from different actors or gathered from relationships enacted within different practice contexts, or even from the same patient at different points in the rehabilitation process. We drew on specific elements of the theories, but, as we used these as sense‐making tools, we did not apply them exhaustively. This means we are touching the surface of what could be considered when analysing data using these theories. Future researchers could explore these, or indeed other, theories further and apply them in greater depth than has been possible in this paper. This reflects that theory is a way to understand practice and there are many options, for example, using feminist theory to delve into how gender impacts on relationships and practice. We do not claim that interpretations we present are generalizable to other contexts, and indeed, the paper did not aim to produce generalizable findings. Instead, we suggest that this paper should encourage SLTs to think critically and reflexively about practice, about relationships with patients, and relationships with colleagues or students, thereby opening up possibilities for enhancing practice.

In conclusion, we believe that our profession benefits from stepping ‘outside the box’ in order to peer back in through a range of different lenses. Therapeutic relationships are fundamental to what we do and achieve with our patients and clients and therefore, we should not take them for granted or only consider them at face value. Our use of analytical pluralism, using theories and critical perspectives in exploring the case of Anna and Olive provides practitioners just one example of how these different lenses might open up new interpretations of everyday interactions and practices, and how these influence therapeutic relationships. Taking time to be reflexive from a micro‐ to a macro‐level has the potential to enrich our practice, teaching and professional growth.
